# Systematic Review and Meta-Analysis of Diagnostic Accuracy of miRNAs in Patients with Pancreatic Cancer

**DOI:** 10.1155/2018/6292396

**Published:** 2018-05-15

**Authors:** Xiao Sun, Xiaobin Zhou, Yuan Zhang, Xiaoyan Zhu, Haihua Liu

**Affiliations:** ^1^Department of Epidemiology and Health Statistics, College of Public Health, Qingdao University, Qingdao, Shandong 266021, China; ^2^Department of Cardiac Surgery, Qingdao Municipal Hospital, Qingdao, Shandong 266021, China

## Abstract

**Background:**

It is reported that miRNAs are aberrantly expressed in patients with pancreatic cancer. However, the diagnostic value of miRNAs in pancreatic cancer remains controversial. The meta-analysis was to access diagnostic accuracy of miRNAs in pancreatic cancer.

**Methods:**

PubMed, Scopus, Web of Science, Chinese National Knowledge Infrastructure (CNKI), WANFANG Data, China Biomedical Literature Database (CBM), and VIP databases were retrieved up to June 30, 2016, to collect articles concerning the diagnosis of miRNAs in pancreatic cancer. The methodological quality of each study was assessed by the Quality Assessment of Diagnostic Accuracy Studies (QUADAS-2). This meta-analysis was conducted using RevMan5.0, MetaDiSc 1.4, and Stata 12.0 software.

**Results:**

There are 40 articles including 109 studies. The pooled SEN was 0.81 (95% CI, 0.80–0.82), the pooled SPE was 0.78 (95% CI, 0.77–0.79), the pooled +LR was 3.32 (95% CI, 2.92–3.80), the pooled −LR was 0.27 (95% CI, 0.24–0.31), the pooled DOR was 14.56 (95% CI, 11.55–18.34), and pooled AUC was 0.86 (95% CI, 0.84–0.88).

**Discussion:**

This meta-analysis demonstrated that miRNA makes a significant impact in the pancreatic cancer diagnosis with a high SEN and SPE, particularly using multiple miRNAs.

## 1. Introduction

Pancreatic cancer (PaC) is one of the most malignant human cancers, with a 5-year survival rate of less than 8% and a survival time of less than 6 months [1, 2]. The surgical resection is an effective treatment for PaC. But the absence of validity for diagnosis at the early stage can lead to low five-year survival rates. The low diagnostic accuracy is caused by insidious onset at the early stage, and the postmortem diagnostics causes a low resection rate and unfavourable prognosis. In addition, PaC and other noncancerous pancreatic diseases (such as chronic pancreatitis (CP)) may show similar symptoms and similar imaging features, which usually lead to erroneous explanation. Therefore, the PaC diagnosis is still a significant clinical challenge. The imaging technologies, such as positron emission tomography (PET), endoscopic ultrasound (EUS), endoscopic ultrasonography, and fine needle aspiration biopsy (FNAB), have high cost and technical difficulty resulting in poor diagnosis [3]. Currently, the most widespread used biomarkers in PaC are CA19-9, CA-125, carcinoembryonic antigen (CEA), MMP-9, K-ras, and p53, but these biomarkers often lead to inadequate specificity (SPE) and unreliable sensitivity (SEN) of PaC and are not recommended for primary screening tools and early disease diagnosis [4]. However, it is a clinically challenging to identify sensitive and specific biomarkers in diagnosis of PaC, especially incipient tumors. Therefore, it has clear clinical significance to develop the effective, credible biomarkers for the early detection and monitoring of PaC.

miRNAs are small noncoding RNA with a length of 18–24 nucleotides, whose main function is to adjust the stability and translation of nuclear mRNA transcripts [5, 6]. A large body of evidence suggests that miRNAs are actively involved in carcinogenesis, as tumor suppressor genes or oncogenes; they have great effects on diagnosis, prognosis, and treatment [7]. Aberrant expression of miRNAs is common in human cancers, including PaC, which are candidate biomarkers for PaC [8]. PaC exhibits higher expression of miR-21, miR-155, miR-146a, miR-196a, miR-196b, miR-200a/b/c, and miR-217. miRNAs are relatively stable in tissues, feces, cyst fluids, plasma, or serum for extraction and test [9, 10]. Hence, the existence of miRNAs may become a biomarker for the early detection of cancer.

Even though the diagnostic accuracy of miRNAs has been confirmed and some studies have achieved promising results, the application of miRNAs in the PaC diagnosis is still disputable and unsatisfactory because of the extensive SEN and SPE values of these studies, which may cause different results dependent on subjects' race, controls' sources, miRNAs' types, and samples tested. For instance, Wang et al. reported 0.64 SEN and 0.89 SPE in Caucasian to determine the diagnostic accuracy of miR-21 [11]. But the results of Liu et al. showed that the diagnostic accuracy of miR-21 for PaC in Asian was 0.71 SEN and 0.69 SPE [12]. Moreover, several studies found that application of single-miRNA profiling for diagnosing PaC performs low diagnostic accuracy. For instance, Liu et al. discovered the value of miR-155 expression as a biomarker for diagnosing PaC in Caucasian. They showed that the sensitivity and specificity of miR-155 expression in plasma were 0.63 and 0.84, respectively, indicating that the accuracy of discriminating pancreatic cancer from chronic pancreatitis was relatively low [13]. However, Ganepola et al. reported a set of miRNAs (miR-642b-3p, miR-885-5p, and miR-22-3p) as biomarkers for the early PaC diagnosis with 0.91 SEN and 0.91 SPE [14]. So far, many studies have confirmed the diagnostic value of miRNAs in PaC [15–17]. However, there is still heterogeneity or inconsistency in the diagnostic accuracy of miRNAs, and its diagnostic value in PaC needs to be confirmed. In view of these discordant results, we performed a meta-analysis to develop the overall diagnostic accuracy of miRNAs in PaC.

## 2. Material and Methods

### 2.1. Search Strategy

**Figure 1:**
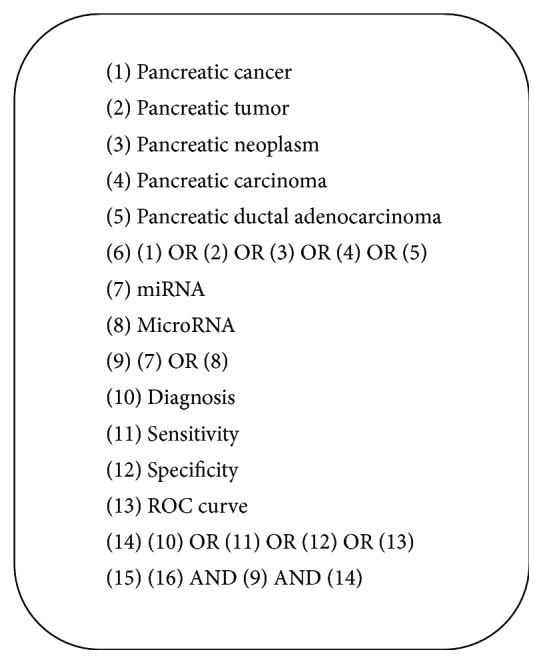
PubMed search strategy.

The documents that met inclusion criteria were identified by searching the following electronic databases: PubMed, Scopus, Web of Science, China Biomedical Literature Database (CBM), Chinese National Knowledge Infrastructure (CNKI), WANFANG Data, and VIP data up to June 30, 2016. The language was limited to Chinese and English. We identified the studies with search terms: “microRNA” or “miRNA”; “pancreatic cancer” or “pancreatic carcinoma” or “pancreatic tumor” or “pancreatic neoplasm” or “pancreatic ductal adenocarcinom” or “pancreatic adenocarcinoma” or “intraductal papillary mucinous neoplasms”; and “diagnosis” or “sensitivity” or “specificity” or “ROC curve”. Combined with Google Scholar and Baidu Scholar, we also scanned the reference lists manually reviewed from included literatures to recognize other relevant studies. The search strategies are shown in Figure 1.

### 2.2. Inclusion and Exclusion Criteria

Eligible studies included in this study must meet the following inclusion criteria: (1) studies concerning the diagnostic value of miRNAs in PaC; (2) sufficient information being reported to construct a four-fold contingency table; and (3) published in English language and Chinese language. The exclusion criteria were as follows: (1) duplicate publications; (2) reviews, abstracts, letters, comments, and case reports; (3) incomplete data to construct a four-fold contingency table; (4) zoopery and other fundamental research; and (5) number of sample in each group < 10.

### 2.3. Data Extraction and Quality Assessment

Two investigators independently evaluated the selected articles. Discrepancies and unobtainable data were resolved by group discussion between at least three investigators. The following data from the included articles was extracted: first author; year of publication; country; subjects' race; total number of cases and controls; source of cases and controls; type of miRNA profiling; and SEN, SPE, true-positive (TP), false-positive (FP), false-negative (FN), and true-negative (TN) values of tested miRNAs. Two authors assessed the risk of bias in each study by using the Quality Assessment of Diagnostic Accuracy Studies (QUADAS-2), which is an efficient tool for evaluating the quality of diagnostic studies [18].

### 2.4. Statistical Methods

The STATA 12.0, RevMan5, and Meta-DiSc 1.4 were used to conduct meta-analysis. We extracted the numbers of all subjects with TP, TN, FP, and FN with their 95% confidence intervals (95% CIs) from each included study. And the pooled SEN, SPE, PLR (positive likelihood ratio), NLR (negative likelihood ratio), and DOR (diagnostic odds ratio) were used for calculation. The summary receiver operating characteristic (SROC) curves were plotted by SEN and SPE, respectively, and the area under the SROC curve (AUC) and 95% CIs were calculated. The AUC shows an analytical summary of test performance and displays the trade-off between SEN and SPE. An AUC of 1.0 (100%) signifies perfect discriminatory ability to distinguish cases from noncases [19]. To evaluate heterogeneity between study, the *Q* test and *I* ^2^ statistics were calculated. *P* < 0.10 for *Q* test or *I* ^2^ value > 50% indicates substantial heterogeneity, and the random effects model was adopted; otherwise, fixed-effects model was adopted [20]. In addition, we also performed subgroup and metaregression analyses to explore potential sources of heterogeneity in the studies. Deeks' funnel plot asymmetry test was used to evaluate publication bias, and *P* < 0.05 was deemed to be statistically significant.

## 3. Results

### 3.1. Literature Search

**Figure 2:**
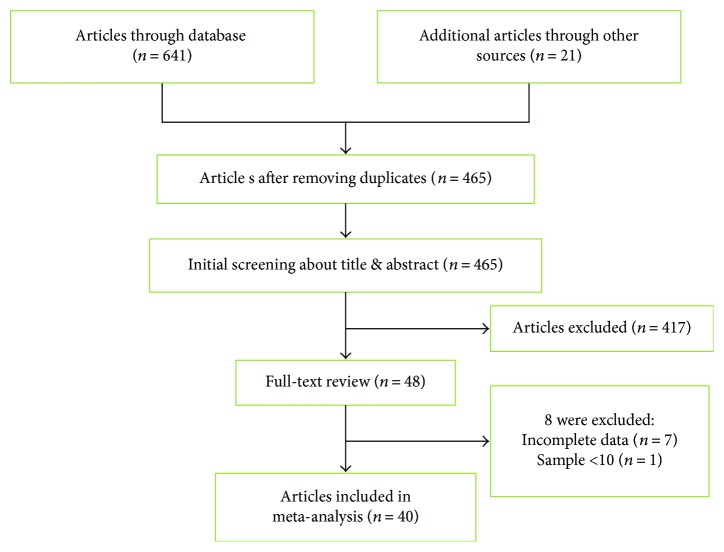
Literature screening process and results. The specific database and the number of retrieved documents are as follows: PubMed (*n* = 67), Web of Science (*n* = 239), Scopus (*n* = 84), CBM (*n* = 142), CNKI (*n* = 56), VIP (*n* = 40), and WANFANG Data (*n* = 13).

The process of literature retrieval was shown in a flow diagram (Figure 2). From electronic databases, we identified 641 potential related studies that used miRNAs for diagnosis in patients with pancreatic cancer and an additional 21 eligible studies included by scanning these documents in our initial study, of which 197 studies were deleted as duplicates. After titles and abstracts were reviewed, 417 studies were further excluded. After reviewing the full-text, we further excluded 8 studies for not about lacking necessary data (*n* = 7), each group contains less than 10 patients (*n* = 1). Ultimately, there are a total of 40 articles for data extraction and analysis.

### 3.2. Characteristic of the Selected Studies

**Figure 3:**
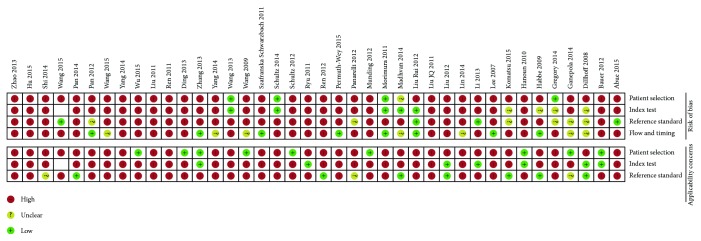
Summary of bias risk assessment results for QUADAS-2.

**Figure 4:**
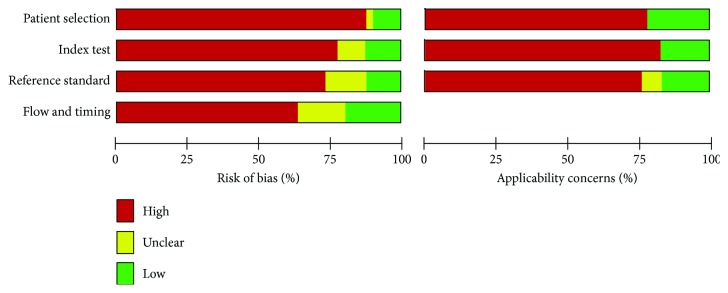
Quality of included studies according to QUADAS-2 guidelines.

The principal characteristics of these included studies were outlined in Table 1. In this study, we found 40 articles, in which 109 studies were conducted for meta-analysis. There were 2878 patients and 2269 controls. Additionally, we showed that 65 studies were conducted in Asian [3, 12, 13, 21–40] and the other 44 studies were conducted in Caucasian [11, 14, 41–53]. There were 80 studies that detected miRNAs in the blood (such as whole blood [14, 46, 53, 54], serum [24, 27, 28, 30, 32, 39, 40, 50, 51], and plasma samples [3, 11–13, 21, 23, 26, 33, 34, 36–38, 52]), and 29 studies detected miRNA in nonblood samples (including tissues [41–43, 45, 47–49, 55], pancreatic juice [44], and stool [22, 25, 31, 35]). We included 71 studies to evaluate the diagnostic efficacy of single miRNAs [12, 13, 21, 22, 26–29, 33, 34, 36, 38, 39, 41–43, 47, 54, 55] and multiple miRNAs [11, 14, 23–25, 30–32, 35, 37, 40, 44–46, 48–53] for distinguishing patients with PaC from controls in 38 studies. The selected studies adopted the reverse transcription polymerase chain reaction (RT-PCR) or immunohistochemistry (IHC) method to detect miRNA expression. The risk of bias and applicability of the studies were evaluated based on QUADAS-2 summarized in Figures 3 and 4.

### 3.3. Diagnostic Accuracy of miRNAs in PaC

**Figure 5:**
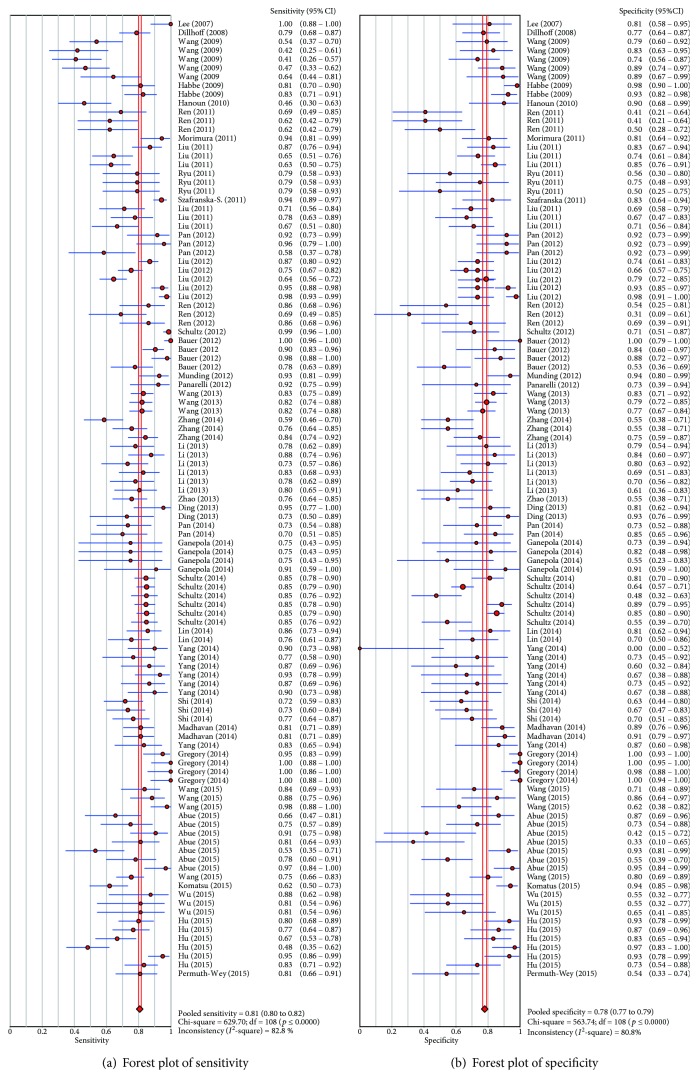
Forest plots of sensitivity (a) and specificity (b) with corresponding heterogeneity statistics for miRNA in pancreatic cancer diagnosis.

By heterogeneity analysis, *I* ^2^ of SEN and SPE was 82.8% (*P* < 0.001) and 80.8% (*P* < 0.001) (Figure 5), respectively, implicating significant heterogeneity of the studies. Thus, the random effects model was applied. To verify whether the heterogeneity could be explained by a threshold effect, we used the Spearman approach for further analysis. Spearman correlation coefficient of these 40 articles was −0.186 (*P* = 0.052), indicating that there was no significant threshold effect.

**Figure 6:**
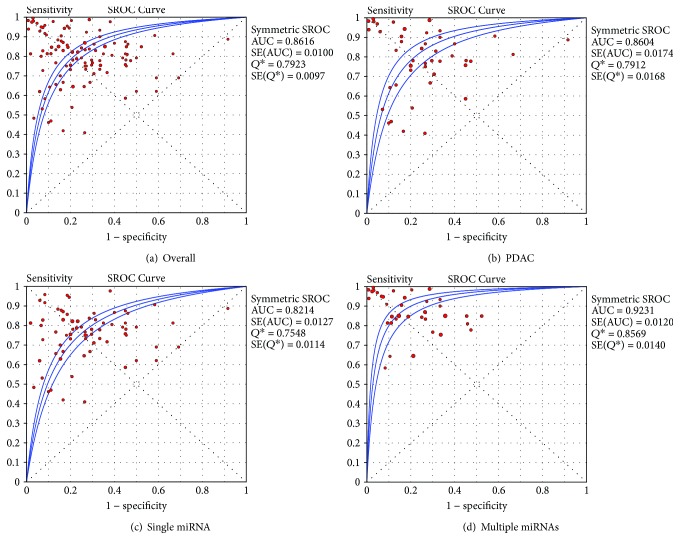
Summary ROC curve with confidence and prediction regions around mean operating sensitivity and specificity point.

The pooled estimates of pancreatic cancer for the diagnostic accuracy of miRNAs were shown in Table 2 and Figure 5. The results were as follows: the pooled SEN was 0.81 (95% CI, 0.80–0.82), the pooled SPE was 0.78 (95% CI, 0.77–0.79), the pooled +LR was 3.32 (95% CI, 2.92–3.801), the pooled −LR was 0.27 (95% CI, 0.24–0.31), and the pooled DOR was 14.56 (95% CI, 11.55–18.34). These results indicated that miRNAs were a valid diagnostic marker for pancreatic cancer. In this meta-analysis, the summary receiver operating characteristic (SROC) results showed that AUC was 0.86, which showed a moderate and perfect level of overall accuracy (Figure 6(a)).

### 3.4. Subgroup Analysis

Considering the significant heterogeneity across studies, we conducted subgroup analysis to explore the source of heterogeneity. The subgroup analysis results were listed in Table 2. Race, patients' types, control source, miRNA profiling, and specimen subgroups showed divergences. Different types of patient subgroup analysis showed that in the 63 studies for the accuracy of miRNAs to distinguish patients with PaC from healthy controls, the pooled SEN was 0.81 (95% CI, 0.79–0.83), the pooled SPE was 0.78 (95% CI, 0.76–0.79), the pooled +LR was 3.42 (95% CI, 2.89–4.05), the pooled −LR was 0.27 (95% CI, 0.23–0.31), the pooled DOR was 14.98 (95% CI, 11.31–19.84), and the AUC was 0.87 (95% CI, 0.84–0.89). Moreover, in the 46 studies for patients with pancreatic ductal adenocarcinoma (PDAC), the pooled SEN was 0.81 (95% CI, 0.79–0.83), the pooled SPE was 0.78 (95% CI, 0.76–0.80), the pooled +LR was 3.19 (95% CI, 2.54–4.02), the pooled −LR was 0.27 (95% CI, 0.21–0.34), the pooled DOR was 14.35 (95% CI, 9.62–21.40), and the AUC was 0.86 (95% CI, 0.82–0.89) (Figure 6(b)). It was suggested that miRNAs had a high diagnostic value in PaC and PDAC. In PDAC, the pooled SEN, SPE, and AUC for 7 studies in which miRNA was measured in tissue were 0.93 (95% CI, 0.90–0.94), 0.85 (95% CI, 0.79–0.90), and 0.93 (95% CI, 0.90–0.94), respectively; the pooled SEN, SPE, and AUC for 30 studies in which miRNA was measured in blood were 0.75 (95% CI, 0.72–0.77), 0.75 (95% CI, 0.72–0.77), and 0.82 (95% CI, 0.81–0.83), respectively; the pooled SEN, SPE, and AUC for 7 studies in which miRNA was measured in feces were 0.87 (95% CI, 0.81–0.91), 0.67 (95% CI, 0.57–0.77), and 0.89 (95% CI, 0.85–0.93), respectively, displaying that miRNA is useful for the diagnosis of PDAC. Due to only 2 studies in pancreatic juice were included, we did not perform a meta-analysis. The DOR in Caucasian populations was higher [20.23, 95% CI (13.61, 30.09)] than Asian populations [11.95, 95% CI (9.05, 15.78)]. Similarly, the AUC in the tissue [0.96, 95% CI (0.93, 0.98)] was higher than the blood [0.85, 95% CI (0.83, 0.87)], but was better than pancreatic juice or fecal miRNAs, revealing that Caucasian populations and detection in the tissue were more accurate in PaC diagnosis. Compared with the detection results with unhealthy controls, detection results from the healthy control group performed a higher level of overall accuracy, indicating that miRNAs were more exact in discriminating patients with PaC from healthy people than from unhealthy people. For the diagnostic accuracy of multiple miRNAs, we found that the AUC was 0.92 (95% CI, 0.89–0.94) (Figure 6(c)); for the diagnostic accuracy of single miRNAs, we found that the AUC was 0.82 (95% CI, 0.79–0.84) (Figure 6(d)), which showed that multiple miRNA profiling is more accurate for the diagnosis of PaC.

### 3.5. Metaregression and Publications Bias

**Figure 7:**
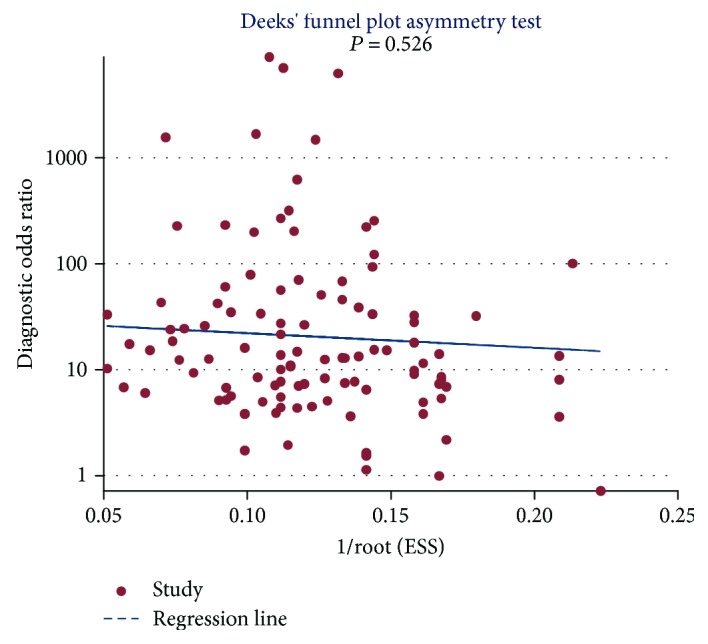
Deeks' funnel plot of miRNA in PaC diagnosis.

A metaregression was conducted to explore the potential heterogeneity within the selected studies. We found that the combination of miR-21 [RDOR = 1.98, 95% CI (1.05, 3.75), *P* = 0.0356] and multiple miRNA profiling [RDOR = 3.07, 95% CI (1.73, 5.44), *P* = 0.0002] was source of interstudy heterogeneity. In this meta-analysis, we also conducted sensitivity analysis to further explore heterogeneity of included studies, which showed that the results of studies were relatively stable and reliable. The Deeks' test performed a statistically nonsignificant value (bias = −3.17, *P* = 0.526) (Figure 7), and the funnel plots were almost symmetric, which showed that there was no potential publication bias.

## 4. Discussion

Pancreatic cancer is a highly malignant tumor with rising incidence and mortality all over the world [56, 57]. The PaC diagnosis remains an urgent clinical challenge, which is due to the relatively absence of symptoms earlier and similar symptoms and imaging features in PaC and other noncancerous pancreatic diseases such as chronic pancreatitis, pancreatic cyst, and IPMN that leads to low accuracy of early diagnosis and incorrect interpretations [58]. miRNAs have clinical potential as diagnostic and predictive markers and as novel molecular targets in PDAC [59]. Jamieson et al. found that expression patterns of miRNAs associated with reduced survival of PDAC, including overexpression of miR-21 and underexpression of miR-34a [60]. Similarly, Frampton et al. focused on meta-analyses that included 1525 patients in PDAC and showed that overall survival OS was significantly shortened in patients with high tumoral miR-21 (adjusted HR = 2.48; 1.96–3.14), indicating that tumoral miR-21 overexpression emerged as an important predictor of poor prognosis in PDAC [61]. Thus, we performed a meta-analysis to access the diagnostic and clinical value of miRNAs as novice biomarkers in PaC.

The conclusions of the current meta-analysis were similar to that of three previous meta-analysis reported by Wan et al. [15], Ding et al. [16], and Pei et al. [17]. Compared with three previous meta-analysis above, there are a number of advantages in the meta-analysis which are as follows: (1) 40 articles with 109 studies are selected in this meta-analysis, which were more studies and participants than three previous meta-analysis; (2) more subgroup analyses than any other reported meta-analysis are performed, especially the article by Wan et al. [15], including race, source of control, miRNA profiling, and the combination of miR-21; and (3) to further demonstrate the potential heterogeneity within the selected studies, our review conducted a metaregression.

In this meta-analyses, we found that the pooled SEN was 0.81 (95% CI, 0.80–0.82), the pooled SPE was 0.78 (95% CI, 0.77–0.79), and the pooled DOR was 14.56 (95% CI, 11.55–18.34), which showed a relatively high level of overall accuracy. LR is widely used for diagnostic criteria of determining or excluding disease [62]. We found that the pooled +LR was 3.32 (95% CI, 2.92–3.801) and the pooled −LR was 0.27 (95% CI, 0.24–0.31). Due to inclusion of early pancreatic cancer in the case group and choosing more healthy subjects in the control group, the diagnostic efficacy of miRNAs was reduced. Therefore, it is necessary to carry out a reasonable design, high-quality, large-sample, prospective study of long-term follow-up to accurately reflect the miRNA diagnostic efficacy. The DOR, which is a diagnostic test evaluation indicator, describes the probability of positive results in patients with the disease compared to the results in patients without disease. In the present analysis, the pooled DOR was 14.56 (95% CI, 11.55–18.34), indicating that patients who tested positive for miRNAs have a 14.56 chances of developing PaC compared to those testing negative, indicating that miRNAs have a higher DOR than the traditional markers in serum such as CEA and CA19-9. Frampton et al. [63] have performed a miRNA metasignature in PDAC and defined a 10-miRNA (upregulated: miR-21, miR-23a, miR-31, miR-100, miR-143, miR-155, and miR-221; downregulated: miR-148a, miR-217, and miR-375) metasignature for PDAC diagnosis.

Due to the significant heterogeneity, we explored potential sources of interstudy heterogeneity, which leads to undermine reliability of study to some degree. It is noteworthy that multiple miRNAs were more accurate in PaC diagnosis than single miRNAs. However, there is no standing panel of valid miRNAs. Yang et al. investigated the combination miR-21, miR-155, and miR-216 with an SEN and an SPE of 0.83 [31]. Liu et al. showed the combination miR-16 and miR-196a with an SEN of 0.87 and an SEN of 0.74 [23]. Ganepola et al. investigated the combination miR-22-3p, miR-642b-3p, and miR-885-5p as markers in early diagnosis PaC with 0.91 SEN and 0.91 SPE [14]. The miR-21 is the most commonly studied. Yang et al. proved that the diagnostic value of miR-21 (SEN: 0.90, SPE: 0.67) was higher than that of miR-216 (SEN: 0.87, SPE: 0.60) [31]. Similarly, Wang et al. performed that miR-21 was more accurate than miR-155 [38]. We also found that miR-21s as biomarkers for the early diagnosis of PaC was more valuable than other miRNAs. In addition, the testing efficiency of miRNAs that were derived from tissue is higher than that of blood and pancreatic juice or feces. The testing threshold plays an important role in disease diagnosis. However, our meta-analyses showed that half of the included studies did not report testing threshold, which may have an impact on the results. Thus, future studies should pay attention to it.

Compared with the conventional biomarkers, miRNAs provide some advantages including sensitive to degradation, more reliable measurement of expression levels, more stable in human specimens, rapid collection, and less invasive [64]. Different levels of miRNA expression help to distinguish between pancreatic cancer patients and healthy controls, which demonstrates potential capability for PaC. Based on the meta-analysis of the 40 articles, we found that miRNAs have a relatively high SEN and SPE in distinguishing patients with PaC from healthy controls, especially using multiple miRNA profiling, which was consistent with the results of Ding et al. [16]. In order to confirm the noninvasiveness, high accuracy, and effectiveness of miRNAs in PaC diagnosis, we still need to further study. For the diagnostic accuracy of miRNAs to distinguish PaC from healthy controls, the SEN was 0.82 (95% CI, 0.81–0.83) and the SPE was 0.76 (95% CI, 0.74–0.78). By contrast, for unhealthy controls, the SEN was 0.79 (95% CI, 0.78–0.81) and the SPE was 0.79 (95% CI, 0.78–0.81), showing a lower accuracy compared with healthy controls. Therefore, we should strictly regulate the diseases among the included studies in the future, especially confusing disease with PaC.

There are limitations when interpreting the results of this study which are as follows: high heterogeneity was conducted in this meta-analysis; the cutoff values are different in the various studies and were not available in some studies; no statistical data was reported concerning African populations; some studies had less sample size; and the vast majority of the studies included healthy subjects as controls.

In conclusion, this meta-analysis showed that miRNA is a useful biomarker for PaC diagnosis and the usefulness of miRNAs in the diagnosis of PaC was pointed out, particularly multiple miRNAs. However, there is still a need for further studies to confirm the validity of employing miRNAs as biomarkers to diagnose PaC.

## Figures and Tables

**Table 1:** The main characteristics of included studies in meta-analysis.

Reference	Country	Ethnicity	Patient spectrum	Source of control	Sample size		miRNA profiling	Specimen	TP	FP	FN	TN	QUADAS-2
					Cases	Controls							
[3]	Japan	Asian	PDAC	DC	32	42	miR-483-3p, miR-21	Plasma	31	2	1	40	8
[41]	USA	Caucasian	PaC	CP + HC	28	21	A set of 9 miRNAs	Tissue	28	4	0	17	8
[42]	USA	Caucasian	PaC	DC	80	57	miR-21	Tissue	63	13	17	44	6
[55]	USA	Caucasian	PaC	HC	64	54	miR-155, miR-21	Tissue	52	1	12	53	8
[11]	USA	Caucasian	PDAC	HC	39	29	miR-21, miR-210, miR-155, miR-196a	Plasma	18	2	10	23	7
[43]	France	Caucasian	PDAC	CP	39	20	miR-148a	Tissue	18	2	21	18	8
[21]	China	Asian	PaC	HC	36	30	miR-18a	Plasma	34	7	2	29	9
[44]	USA	Caucasian	PaC	HC	24	16	miR-21, miR-221, miR-17-3p	Pancreatic juice	19	4	5	12	10
[45]	South America Spain Middle East	Caucasian	PDAC	DC	157	29	A set of 7 miRNAs	Tissue	148	5	9	24	8
[13]	China	Asian	PaC	CP + HC	62	97	miR-155	Plasma	39	15	23	82	7
[12]	China	Asian	PDAC	CP + HC	45	75	miR-21	Plasma	32	23	13	52	6
[22]	China	Asian	PaC	CP	29	22	miR-181b, miR-196b, miR-210	Fecal	20	13	9	9	7
[46]	Germany	Caucasian	PDAC	CP	45	38	A set of 100 miRNAs	Blood	35	18	10	20	7
[24]	China	Asian	PaC	CP + HC	138	175	miR-16, miR-196a	Plasma	89	37	49	138	8
[23]	China	Asian	PaC	CP + HC	95	81	A set of 7 miRNAs	Serum	90	6	5	75	8
[47]	Germany	Caucasian	PDAC	CP	42	33	miR-135b	Tissue	39	2	3	31	6
[48]	USA	Caucasian	PDAC	DC	26	11	miR-21, miR-221, miR-155, miR-100, miR-196a, miR-181b	Tissue	24	3	2	8	9
[25]	China	Asian	PaC	HC	29	13	miR-181b, miR-210, miR-196a	Fecal	25	6	4	7	7
[49]	Denmark	Caucasian	PDAC	HC	160	28	A set of 9 miRNAs	Tissue	158	8	2	20	6
[26]	China	Asian	PaC	HC	24	24	miR-451, miR-409-3p	Plasma	22	2	2	22	6
[50]	USA	Caucasian	PDAC	CP	41	35	miR-1290, miR-146a	Serum	34	11	7	24	7
[27]	China	Asian	PaC	DC	129	163	miR-27a-3p	Blood	106	34	23	129	7
[28]	China	Asian	PDAC	HC	70	40	miR-192	Serum	53	18	17	22	5
[29]	China	Asian	PaC	DC	22	27	miR-21, miR-17-5p	Serum	21	5	1	22	8
[51]	Germany	Caucasian	PaC	DC	75	53	miR-1246, miR-4644, miR-3976, miR-4306	Serum	61	5	14	48	7
[14]	USA	Caucasian	PaC	HC	11	11	miR-885-5p, miR-22-3p, miR-642b-3p	Blood	10	1	1	10	5
[52]	USA	Caucasian	PDAC	DC	40	54	A set of 5 miRNAs	Plasma	38	0	2	54	8
[30]	China	Asian	PaC	HC	49	27	miR-492, miR-663a	Serum	42	5	7	22	6
[53]	Denmark	Caucasian	PaC	HC	180	199	A set of 10 miRNAs	Blood	153	29	27	170	8
[35]	China	Asian	PDAC	HC	30	15	miR-21, miR-155, miR-216	Fecal	28	5	2	10	8
[32]	China	Asian	PDAC	HC	70	40	miR-192, miR-194	Serum	59	10	11	30	7
[33]	China	Asian	PaC	HC	30	26	miR-210, miR-25	Plasma	22	7	8	19	6
[34]	China	Asian	PaC	CP + HC	60	30	miR-155, miR-196a	Plasma	46	9	14	21	6
[31]	China	Asian	PDAC	HC	30	15	miR-21, miR-155, miR-216	Fecal	25	2	5	13	6
[36]	Japan	Asian	PaC	HC	71	67	Circulating miR-223	Plasma	44	4	27	63	7
[54]	USA	Caucasian	PaC	HC	42	24	A set of 30 miRNAs	Plasma	34	11	8	13	7
[37]	China	Asian	PaC	CP + HC	60	30	miR-155, miR-21, miR-196a, miR-210	Plasma	57	2	3	28	6
[38]	China	Asian	PaC	HC	43	21	miR-21, miR-155	Plasma	38	3	5	18	5
[39]	China	Asian	PDAC	CP	110	70	miR-155	Serum	83	14	27	56	8
[40]	China	Asian	PaC	DC	16	20	miR-21, miR-29a, miR-210	Serum	13	9	3	11	6

PaC: pancreatic cancer; PDAC: pancreatic ductal adenocarcinoma; HC: healthy control; CP: chronic pancreatitis; DC: disease control; TP: true positive; FP: false positive; FN: false negative; TN: true negative.

**Table 2:** Summary estimates of subgroup analysis for miRNA in pancreatic cancer diagnosis.

Subgroups	Number of studies	SEN (95% CI)	SPE (95% CI)	+LR (95% CI)	−LR (95% CI)	DOR (95% CI)	AUC (95% CI)
Source of patients							
PDAC	46	0.81 (0.79, 0.83)	0.78 (0.76, 0.80)	3.19 (2.54, 4.02)	0.27 (0.21, 0.34)	14.35 (9.62, 21.40)	0.86 (0.82, 0.89)
PaC	63	0.81 (0.79, 0.82)	0.78 (0.76, 0.79)	3.42 (2.89, 4.05)	0.27 (0.23, 0.31)	14.98 (11.31, 19.84)	0.87 (0.84, 0.89)
Source of control							
Healthy control	58	0.82 (0.81, 0.83)	0.76 (0.74, 0.78)	3.21 (2.66, 3.87)	0.26 (0.22, 0.31)	14.29 (10.41, 19.60)	0.86 (0.83, 0.88)
Disease control	51	0.79 (0.78, 0.81)	0.79 (0.78, 0.81)	3.46 (2.84, 4.23)	0.28 (0.23, 0.33)	14.96 (10.63, 21.05)	0.86 (0.83, 0.89)
Ethnicity							
Asian	65	0.78 (0.77, 0.80)	0.76 (0.74, 0.78)	3.02 (2.55, 3.57)	0.30 (0.26, 0.34)	11.95 (9.05, 15.78)	0.84 (0.81, 0.86)
Caucasian	44	0.84 (0.82, 0.85)	0.80 (0.78, 0.82)	3.91 (3.10, 4.92)	0.23 (0.18, 0.29)	20.23 (13.61, 30.09)	0.88 (0.85, 0.91)
miRNA profiles							
Single miRNA	71	0.75 (0.74, 0.77)	0.76 (0.74, 0.77)	2.82 (2.41, 3.29)	0.34 (0.30, 0.39)	9.48 (7.44, 12.08)	0.82 (0.79, 0.84)
Multiple miRNAs	38	0.87 (0.86, 0.88)	0.81 (0.79, 0.82)	4.65 (3.62, 5.97)	0.16 (0.13, 0.21)	35.88 (22.92, 56.16)	0.92 (0.89, 0.94)
Source of miRNA							
Blood	80	0.79 (0.78, 0.80)	0.78 (0.76, 0.79)	3.35 (2.92, 3.84)	0.29 (0.25, 0.33)	13.64 (10.74, 17.33)	0.85 (0.83, 0.87)
Tissue	13	0.90 (0.88, 0.92)	0.90 (0.87, 0.92)	7.14 (4.43, 11.50)	0.09 (0.05, 0.19)	96.13 (39.20, 235.77)	0.96 (0.93, 0.98)
Fecal	13	0.80 (0.76, 0.84)	0.57 (0.49, 0.63)	1.77 (1.28, 2.44)	0.35 (0.23, 0.55)	5.52 (2.67, 11.42)	0.75 (0.66, 0.85)
Pancreatic juice	3	0.79 (0.68, 0.88)	0.60 (0.45, 0.74)	1.87 (1.31, 2.68)	0.34 (0.21, 0.57)	5.77 (2.53, 13.15)	0.76 (0.66, 0.85)
miRNA-21							
Yes	24	0.85 (0.83, 0.87)	0.83 (0.80, 0.86)	4.76 (2.96, 7.66)	0.19 (0.13, 0.28)	30.24 (15.44, 59.21)	0.91 (0.87, 0.95)
No	85	0.80 (0.78, 0.81)	0.77 (0.75, 0.78)	3.06 (2.67, 3.50)	0.29 (0.25, 0.33)	12.31 (9.69, 15.65)	0.84 (0.82, 0.86)
Overall	109	0.81 (0.80, 0.82)	0.78 (0.77, 0.79)	3.32 (2.90, 3.80)	0.27 (0.24, 0.31)	14.56 (11.55, 18.34)	0.86 (0.84, 0.88)

SEN: sensitivity; SPE: specificity; +LR: positive likelihood ratio; −LR: negative likelihood ratio; DOR: diagnostic odds ratio; AUC: area under the curve; PDAC: pancreatic ductal adenocarcinoma.

## Supplementary Materials

Supplementary Material The database consists of the following data selected articles.
